# Awareness of Colorectal Cancer and Attitudes Towards Its Screening Guidelines in Lebanon

**DOI:** 10.5334/aogh.2437

**Published:** 2019-05-28

**Authors:** Mohamad Ali Tfaily, Dana Naamani, Alaa Kassir, Sara Sleiman, Mamadou Ouattara, Munir Paul Moacdieh, Miran A. Jaffa

**Affiliations:** 1 Faculty of Medicine, American University of Beirut, Beirut, LB; 2 Department of Research Training and Communication, Nouna Health Research Center (CRSN), Nouna, BF; 3 Epidemiology and Population Health Department, Faculty of Health Sciences, American University of Beirut, Beirut, LB

## Abstract

**Background:**

Screening for colorectal cancer (CRC) provides an effective strategy for early detection and prevention of the disease; however, global screening rates are still low.

**Purpose:**

This study aims at assessing the awareness of CRC risk factors, warning signs, and attitudes towards CRC guidelines and screening modalities, in order to identify the barriers to and correlates of CRC screening in the Lebanese population.

**Methods:**

A self-administered questionnaire was distributed to 371 participants in the largest health care medical center in Lebanon. A validated 12- and 9-item Cancer Awareness Measurement questionnaire was used to assess participants’ awareness of CRC risk factors and warning signs.

**Results:**

83% and 67% of participants were not aware of CRC risk factors and warning signs, respectively, 15% have previously undergone CRC screening, 56% were aware of the necessity for screening, and 43% were willing to undergo screening. Factors affecting awareness of the necessity for CRC screening, past screening and willingness to screen included awareness of risk factors and warning signs, undergoing regular physician check-ups, having a family physician as a primary source of knowledge of CRC, and knowing a family member or friend diagnosed with CRC. Barriers to screening were related to participants’ evaluation of the screening technique and misconceptions about this disease.

**Conclusion:**

Serious active measures should be taken by health care sectors, authoritative groups, primary care physicians, and awareness campaigns to fill the gap in awareness of this disease and to alleviate the barriers and misconceptions around it.

## Introduction

Colorectal cancer (CRC) is one of the most prevalent cancers in the world. It is the second most common cancer in women and the third most common cancer in men. CRC-associated mortality is also elevated, reaching an estimated 551,269 deaths in 2018. The incidence per 100,000 of colorectal cancer varies widely across the Middle East and North Africa region, from a 6.6 in Egypt to a 32.0 in Jordan. Lebanon has one of the highest incidence rates in the region, with 1109 cases of CRC diagnosed in 2018, making it the second most common cancer among Lebanese women and fourth among Lebanese men [1,2]. CRC screening techniques play a crucial role in decreasing the burden of this disease by allowing the excision of premalignant adenomas before the emergence of cancer, thereby increasing chances of survival [3]. Therefore, screening for CRC is imperative and recommended for all individuals above the age of 50. Particularly, it is recommended that all individuals get screened by colonoscopy once every 10 years, or by sigmoidoscopy once every five years. Individuals without a family history of CRC are recommended to start screening at the age of 50, whereas individuals with a positive family history of CRC should start screening earlier at the age of 40 [4]. Recently, the American Cancer Association updated its screening guidelines, lowering the age at which screening for colorectal cancer should begin to 45 years old for average risk individuals [5].

In the USA, awareness about CRC screening is on the rise and screening rates have reached a stable 60% of the US population. However, screening rates vary among different subpopulations, in correlation with diverse criteria like ethnicity and level of education [6]. There are numerous studies assessing the awareness of the public about CRC screening and the different factors that may influence the compliance to screening within given populations. These factors range from populational criteria that correlate with higher or lower levels of compliance to screening, to criteria that are self-reported to affect one’s screening habits. To our knowledge, only one study was conducted in Lebanon that examined awareness of signs, risk factors and screening methods of colorectal cancer, but no studies addressed the attitudes of the Lebanese population towards colorectal cancer screening. The study by Nemer et al. [3] assessed awareness of CRC in Lebanon and established that a very low proportion of CRC-aware respondents showed willingness to undergo screening for CRC. These findings give urgency to explore possible barriers to CRC screening that may be negatively impacting individuals’ attitudes towards CRC and their willingness to get screened.

The objective of this study is to assess the awareness of the Lebanese population to the warning signs and risk factors of colorectal cancer, and examine the attitudes and barriers towards CRC screening guidelines and modalities in Lebanon. We also aim to determine if awareness of CRC risk factors and warning signs correlates with better attitude towards the necessity of and willingness to screen. Ultimately, this will help provide guidance for future efforts to improve CRC screening rates, early detection and disease prevention.

## Methods

*Study design, setting and sampling*: This is a cross-sectional study that employed a self-administered questionnaire wherein information was collected on awareness of and attitudes towards CRC and its screening protocol, barriers associated with screening modalities, and awareness of warning signs and risk factors for CRC. The study has been conducted at the American University of Beirut Medical Center (AUBMC), one of the largest health care centers in Lebanon that hosts patients from all over the country, making the participants and our sample a good representative of the Lebanese population. Our target population included Lebanese citizens that are at least 25 years old, residing in Lebanon, and with no history of CRC, inflammatory bowel disease, or polyps. Immigrants and citizens who do not reside in Lebanon were excluded from the study. The self-administered questionnaire was distributed to random participants at AUBMC in various waiting rooms in clinics and outpatient departments, main entrances and resting areas. The study protocol was approved by AUB’s Institutional Review Board. Participants were asked for a signed oral consent before their enrolment in the study. Our sample size was determined using power analysis from the regression approach that assumes a medium effect size of 0.1, number of covariates of 20, significance levels of 0.05, and power of 80%. This resulted in a sample size of about 340. To account for missing data and non-response we inflated our study by 1.1 times and increased the sample size to 371 participants randomly and equally selected from the locations stated above.

*Survey instrument and variables*: Three major outcomes were considered in the regression models which included past CRC screening, attitude towards future CRC screening, and awareness of necessity of CRC screening. The correlations between these outcomes and awareness of CRC warning signs and risk factors along with other covariates were examined. Questions on the awareness of CRC risk factors and warning signs were determined using the relevant modules from Cancer Awareness Measurement, UK (CAM). “This survey instrument (Bowel CAM) was developed by University College London and Cancer Research UK. It is based on a generic CAM developed by Cancer Research UK, University College London and Oxford University in 2007–2008 [7].” To assess the barriers, two studies in the literature Wong et al. and Klabunde et al. [8,9] were used as a reference to develop the questions and scales in our questionnaire. We have assessed 4 factors: awareness of risk factors of CRC, awareness of the symptoms of CRC, awareness of CRC screening protocol, and barriers towards completing the screening tests for CRC. The CAM questionnaire on awareness of risk factors and warning symptoms of CRC was translated to Arabic and validated before being used in our study as described in the supplementary section (Appendices 1 and 2). The validation result showed high overall percent agreement of 95% between the Arabic and English CAM questionnaires. The CAM survey instrument used for assessing the level of awareness of CRC risk factors consisted of a set of multiple choice questions wherein participants were given a set of risk factors in the form of 12 questions that are related to CRC, and asked to choose between “yes”, “no”, and “I don’t know” for each risk factor, where “yes” was the correct answer. If all questions were answered correctly, then this gives the participants a perfect score of 12 over 12. Similarly, awareness of warning signs included 9 relevant questions and if all questions were answered correctly then a perfect score of 9 over 9 would be obtained. Moreover, participants were also asked about awareness of screening protocols, like colonoscopy and fecal occult blood test (FOBT), their attitudes towards CRC screening and barriers that hindered their participation in such tests. The covariates considered in our study included personal information such as age, gender, marital status, employment, occupational sector, income, checkups with general practitioner, insurance coverage, educational level: (Highest educational level achieved), smoking (yes/no, frequency), drinking (yes/no, frequency), encountering a CRC patient, and demographics.

*Statistical Analysis*: We initiated our analysis by identifying any outliers or data entry errors. Descriptive analysis was conducted to provide a summary of the characteristics of our study population and response data on the CRC awareness presented graphically and numerically in terms of frequencies and percentages. Logistic regressions were conducted to determine the associations in terms of odds ratios (ORs), their 95% confidence intervals, and P-values, between the different covariates and the outcomes of interest described above. A cumulative knowledge score for the risk factors (range 0–12) was obtained by summing up the correct answers to the 12-item questionnaire. A cumulative score of 9 or above implied that the participant was aware of the risk factors of CRC, whereas any score lower than 9 would indicate insufficient awareness. Similarly, the cumulative knowledge score for CRC warning signs (range 0–9) was calculated and a score of 7 or above indicated awareness of CRC warning signs, whereas any score lower than 7 implied insufficient awareness. This grading was done according to the Health Belief Model and the guidelines set by CAM, as used in the study by Bidouei et al. [10] Analysis was performed with a level of significance of 0.05 using SPSS version 24, and STATA version 13.

## Results

*Demographics*: Our sample was comprised of 371 participants surveyed between the months of March and April, 2018. The demographic distribution of our participants is shown in Table 1. Missing data was due to missing responses on some questions and accounted for less than 10% of our overall sample. 39% of the participants were 25 to 40 years old, and 59% were above the age of 40 inclusive. Participants had almost equal distribution in gender, higher education, and regular physician check-ups. More than half of the participants (66%) did not know of any family member or friends with CRC, had insurance coverage (78%), and were currently employed (65%) with a job not related to health care (83.6%).

**Table 1:** Demographics of study participants.

Characteristic	No.	%

**Age groups**		
25 to 40	145	39
40 to 50	86	23.1
Above 50	134	36
**Sex**		
Male	161	43.3
Female	181	48.7
**Education**		
University degree level	206	56.6
Below university degree level	158	43.4
**Job related to health care**		
No	301	83.6
Yes	59	16.4
**Currently employed**		
No	123	33.1
Yes	244	65.6
**Monthly Income**		
Below middle income ($2000 or less)	164	48.8
Above middle income (more than $2000)	71	21.1
Prefer not to say	101	30.1
**Insurance**		
No	79	21.5
Yes	288	78.5
**Regular physician check-ups**		
No	181	49.5
Yes	183	50.0
I don’t know	2	0.5
**Family or close friends had CRC history**		
No	246	66.1
Yes	105	28.2
I don’t know	16	4.3

*Awareness of risk factors and symptoms of CRC*: Our results showed that only 17.2% of the participants were aware of CRC risk factors while 31.5% were aware of CRC symptoms. Participants’ awareness of CRC risk factors was presented in Table 2. The results indicated that a high percentage (51.1%) of participants erroneously thought that CRC is not related to age and only 31.6% were correctly aware that age of 60 years puts the individual at a higher risk of CRC compared to other ages. A high percentage of participants erroneously thought that absence of physical activity (38.7%) and having diabetes (41.6%) are not risk factors for CRC. As for the participants’ awareness of warning signs of CRC, over 50% of the participants did not recognize that anemia and fatigue are symptoms of CRC (Table 3). Our unadjusted logistic regression analysis showed that knowing people with CRC doubled the odds of being aware of CRC risk factors (OR = 1.787, 95% CI for OR = [1.017, 3.139], and P-value = 0.043), and CRC warning signs (OR = 2.176, 95% for OR = [1.357, 3.491], and P-value = 0.001).

**Table 2:** Awareness of the risk factors of CRC as per the awareness module of the CAM questionnaire.

Risk Factors	No.	%

**Age**		
20-year-old	4	1.1
40-year-old	55	15.1
60-year-old (correct answer)	115	31.6
Unrelated to age	186	51.1
I don’t know	4	1.1
**Drinking alcohol**		
Yes	191	52.8
No	73	20.2
I don’t know	98	27.1
**Not eating fruits and vegetables**		
Yes	157	42.7
No	130	35.3
I don’t know	81	22.0
**Eating red meat**		
Yes	237	64.4
No	67	18.2
I don’t know	64	17.4
**Low fiber diet**		
Yes	208	56.7
No	72	19.6
I don’t know	87	23.7
**Having a family member with CRC**		
Yes	195	53.6
No	103	28.3
I don’t know	66	18.1
**Being 70 years old**		
Yes	155	42.5
No	101	27.7
I don’t know	109	29.9
**Absence of physical activity**		
Yes	131	36.5
No	139	38.7
I don’t know	89	24.8
**Presence of bowel disease**		
Yes	221	62.1
No	49	13.8
I don’t know	86	24.2
**Having diabetes**		
Yes	94	26.0
No	150	41.6
I don’t know	117	32.4
**Smoking**		
Yes	217	60.8
No	75	21.0
I don’t know	65	18.2
**Obesity**		
Yes	213	58.0
No	66	18.0
I don’t know	88	24.0

**Table 3:** Awareness of warning signs of CRC as per the CAM questionnaire.

Risk Factors	No	%

**Anal bleed**		
Yes	249	68.2
No	36	9.8
I don’t know	80	22
**Pain in abdomen**		
Yes	225	61.6
No	68	18.6
I don’t know	72	19.7
**Change in bowel habits**		
Yes	205	56.3
No	74	20.3
I don’t know	85	23.3
**Bowel not emptying**		
Yes	151	42.1
No	94	26.2
I don’t know	114	31.7
**Blood in your stools**		
Yes	237	65.3
No	54	14.9
I don’t know	72	19.8
**Pain in your back passage**		
Yes	167	46
No	83	22.9
I don’t know	113	31.1
**Lump in anus**		
Yes	212	58.6
No	58	16
I don’t know	92	25.4
**Anemia/fatigue**		
Yes	143	39.3
No	104	28.6
I don’t know	117	32.1
**Unexplained weight loss**		
Yes	216	59.2
No	54	14.8
I don’t know	95	26

*Awareness of the necessity of CRC screening*: 55% of participants were aware that they should screen for colorectal cancer. Results in Table 4 from multiple logistic regression analysis showed that participants who were aware of CRC risk factors had double the odds of being aware of the necessity of CRC screening (OR = 2.221, 95% CI = [1.023, 4.820], and P-value = 0.04). Similarly, older participants (above 50 years of age) and those with family physician as primary source of knowledge about cancer had twice the odds of being aware of the need for CRC screening ([OR = 2.376, 95% CI for OR = (1.362, 4.147), and P-value = 0.002]; [OR = 2.384, 95% CI for OR = (1.209, 4.700), and P-value = 0.012] respectively). Moreover, those who undergo regular physician check-ups had triple the odds of being aware of the necessity of CRC screening (OR = 3.167, 95% CI for OR = [1.884, 5.323], and P-value <0.0001). University versus non-university level of education, and all other remaining covariates listed in Table 4 failed to have significant associations with the awareness of the necessity of CRC screening.

**Table 4:** Multiple logistic regression showing adjusted associations between below factors and awareness of necessity of CRC screening.

	95% Confidence Interval for Odds Ratio			

Factors	Odds Ratio	Lower Limit	Upper Limit	P-value

Sex (Reference male)	1.607	0.934	2.765	0.087
Family or close friends had CRC history	1.454	0.938	2.253	0.095
Risk Factors Awareness	2.221	1.023	4.820	0.044
Warning Signs Awareness	1.196	0.670	2.134	0.545
Age above 50 years	2.376	1.362	4.147	0.002
Level of Education	0.777	0.454	1.332	0.359
Smoking Status	0.897	0.542	1.485	0.673
Regular Physician Check-Ups	3.167	1.884	5.323	0.000
Employment Status	1.640	0.885	3.041	0.116
Job related to healthcare	1.022	0.492	2.123	0.953
Method of awareness about cancer (family doctor)	2.384	1.209	4.700	0.012
Method of awareness about cancer (TV)	0.613	0.355	1.059	0.079

*Predictors of past CRC screening*: 15% of participants reported that they had screened for CRC before. Results of the multiple logistic regression with outcome being the occurrence of at least one CRC screening in the past are presented in Table 5. Participants above the age of 50 years had 4 times the odds of undergoing CRC screening in the past (OR = 4,223, 95% for OR = [1.708, 10.442], and P-value = 0.002) compared to those who were less than 50 years old. Those with a family member or friend diagnosed with CRC had triple the odds of undergoing CRC screening themselves (OR = 3.364, 95% for OR = [1.452, 7.795], and P-value = 0.005) compared to those who never knew any friend or family member with this disease. Our results also indicated that those who were aware of CRC warning signs had double the odds of undergoing previous CRC screening (OR = 2.565, 95% for OR = [1.050, 6.266], and P-value = 0.039) compared to those who were not aware of these signs.

**Table 5:** Multiple logistic regression showing adjusted associations between below factors and undergoing past CRC screening.

	95% Confidence Interval for Odds Ratio			

Factors	Odds Ratio	Lower Limit	Upper Limit	P-value

Lived in a different country	1.147	0.467	2.818	0.765
Sex (Reference male)	0.570	0.229	1.416	0.226
Marital Status	0.727	0.232	2.276	0.584
Job Related to Healthcare	1.097	0.341	3.536	0.876
Insurance or NSSF	2.586	0.765	8.742	0.126
Family or Close Friends Having CRC	3.364	1.452	7.795	0.005
Warning Sign Awareness	2.565	1.050	6.266	0.039
Risk Factor Awareness	0.556	0.203	1.523	0.254
Age above 50 years	4.223	1.708	10.442	0.002
Employment Status	0.403	0.152	1.070	0.068
Level of Education	0.651	0.263	1.611	0.353
Smoking Status	0.880	0.376	2.062	0.769
Regular Physician Check-Ups	2.059	0.762	5.563	0.155

**Figure 1:**
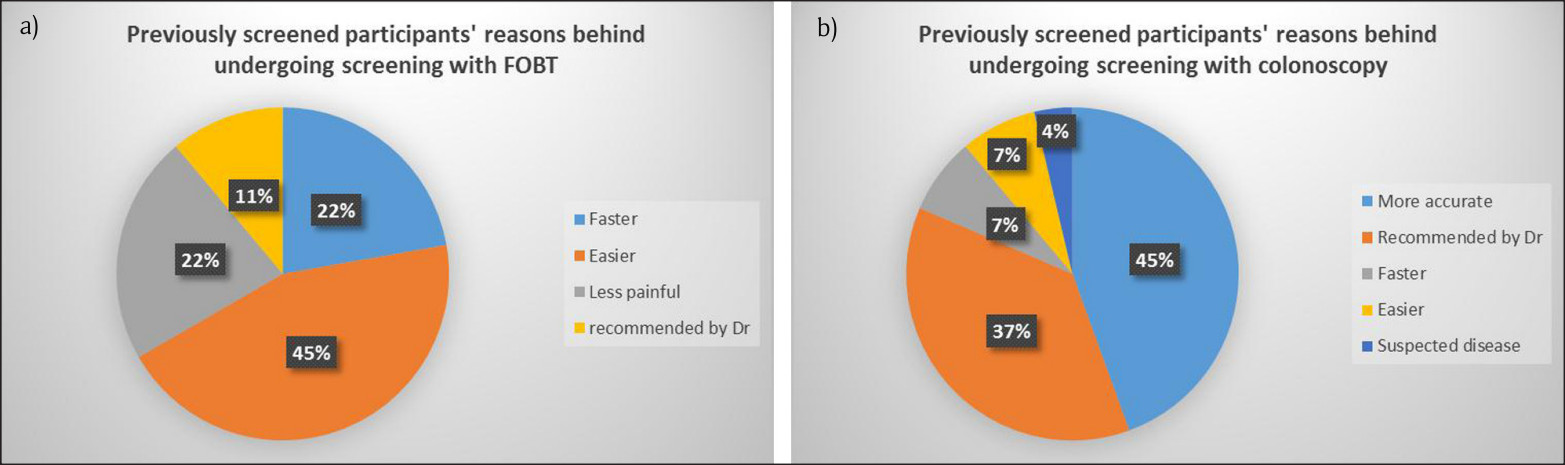
Previously screened participants’ reasons for choosing **a)** FOBT over Colonoscopy and **b)** Colonoscopy over FOBT.

*Attitudes towards CRC screening among participants who have been previously screened*: The majority (87%) of respondents who were previously screened for CRC had chosen colonoscopy over FOBT. The reasons reported for choosing FOBT over colonoscopy as screening method in the past are shown in Figure 1a, and those for choosing colonoscopy over FOBT are depicted in Figure 1b. Forty-five percent of those who underwent previous CRC screening considered FOBT to be faster than colonoscopy (Figure 1a), and 45% viewed colonoscopy as a more accurate screening test (Figure 1b).

Among individuals who had already gotten screened for CRC, 77.3% reported that they were planning to get screened again in the future. The majority of the remaining 22.7% who were not planning to get screened again in the future related their decision to the reasons that CRC screening was uncomfortable (40%), painful (20%), time consuming (10%), or embarrassing (10%). Reasons for not planning to get screened in the future were not mutually exclusive and the ones listed here were the ones that recurred the most.

*Attitudes towards CRC screening among participants who have never screened before*: Among participants who had not been screened to date, 43% reported willingness to get screened in the future, while 44% denied planning to get screened and the remaining 13% were unsure. Table 6 shows the association between different variables and willingness to screen in the future for participants who have not been screened before. Undergoing regular physician check-ups and being aware of CRC risk factors had double the odds of willing to screen for CRC in the future with (OR = 2.477, 95% for OR = [1.430, 4.291], and P-value = 0.001), and (OR = 2.641, 95% for OR = [1.217, 5.732], and P-value = 0.014) respectively.

**Table 6:** Multiple logistic regression showing adjusted associations between below factors and willingness to undergo CRC screening in the future among those who have never been previously screened.

	95% Confidence Interval for Odds Ratio			

Factors	Odds Ratio	Lower Limit	Upper Limit	P-value

Sex (Reference male)	0.960	0.548	1.682	0.886
Marital Status	1.329	0.693	2.548	0.393
Job related to healthcare	0.874	0.412	1.855	0.726
Insurance or NSSF	0.601	0.307	1.175	0.136
Regular Physician Check-Ups	2.477	1.430	4.291	0.001
Employment status	1.124	0.588	2.147	0.724
Level of Education	1.256	0.694	2.273	0.451
Smoking Status	0.827	0.482	1.419	0.490
Risk Factor Awareness	2.641	1.217	5.732	0.014
Warning Sign Awareness	0.889	0.472	1.676	0.717
Age above 50 years	0.891	0.484	1.639	0.710
Knowing family/friends with CRC	1.364	0.748	2.488	0.311
Lived in a different country	0.972	0.531	1.780	0.927

**Figure 2:**
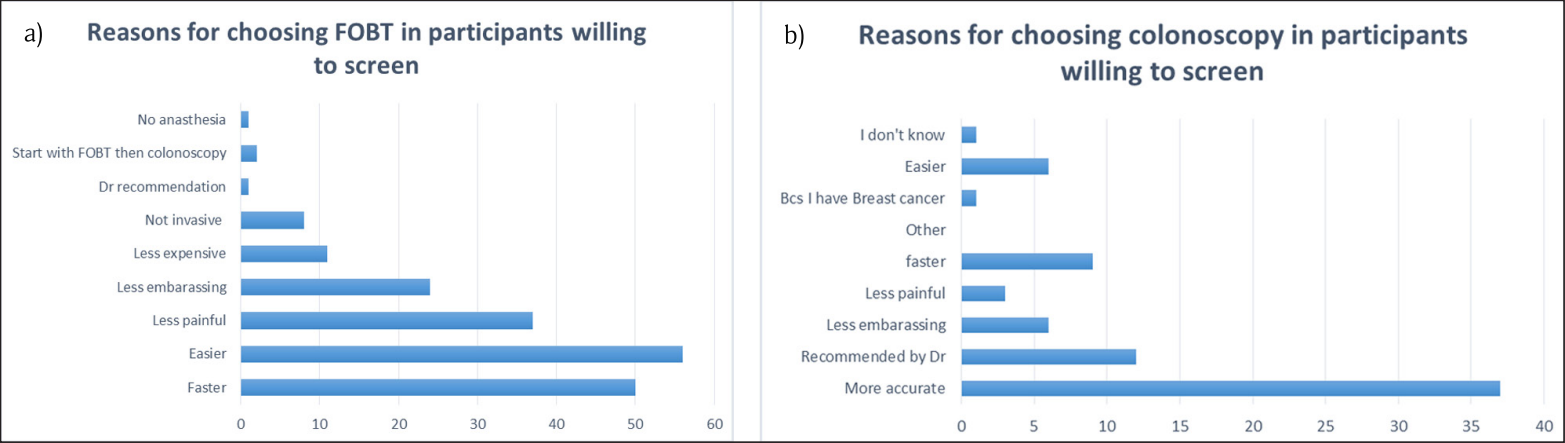
Reasons for choosing **a)** FOBT **b)** Colonoscopy among previously unscreened participants who are willing to undergo screening in the future.

**Figure 3:**
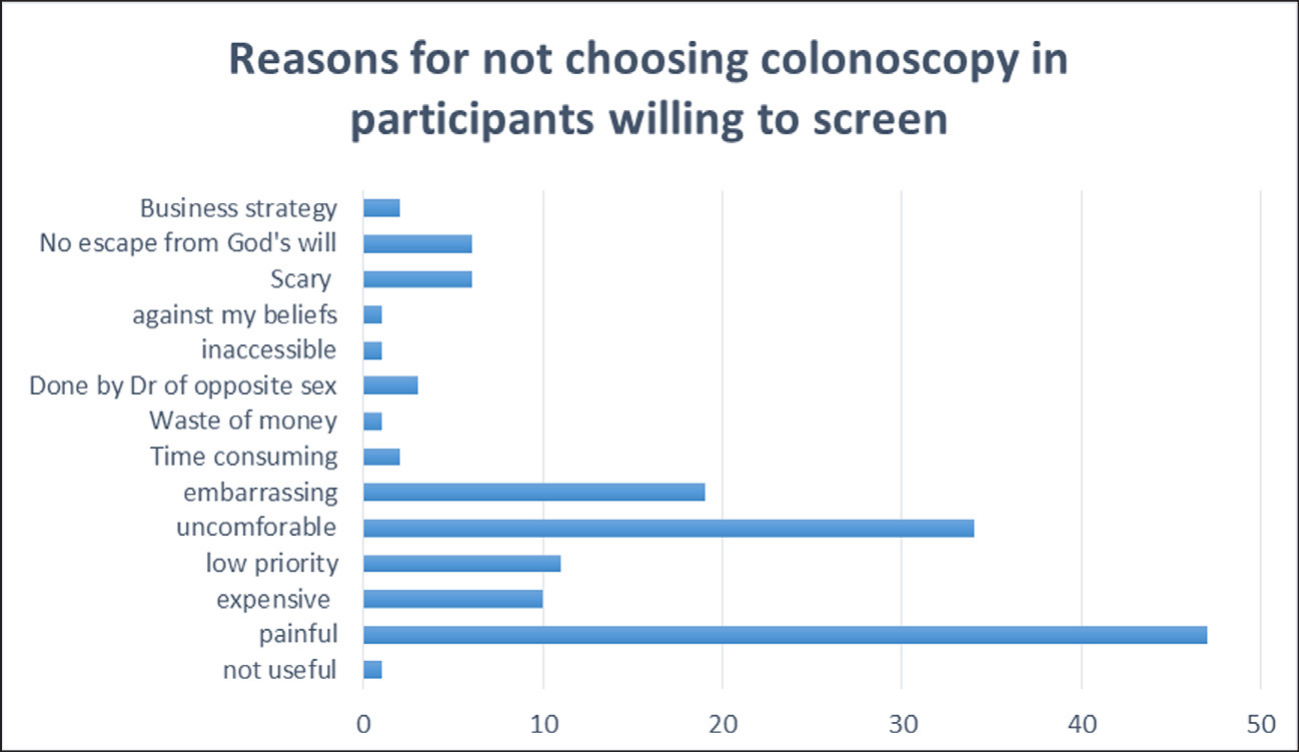
Reasons for not choosing Colonoscopy among previously unscreened participants who are willing to undergo screening in the future.

50% of patients who were planning to get screened in the future selected FOBT as their preferred method of screening and 42% preferred colonoscopy (the remaining 8% chose neither or no preference). Being easier than colonoscopy was the most prominent reason for preferring FOBT (Figure 2a), while accuracy was the main reason for preferring colonoscopy (Figure 2b). The belief that colonoscopy was painful was the main barrier that precludes participants from selecting it in the future for CRC screening (Figure 3).

In addition, 34% of participants who were unwilling to screen for CRC in the future reported that there was no need to screen since they do not have anyone in the family with CRC, 40% of them considered it as low priority, and 17% thought that there is no escape from God’s will. These were the most common reasons for not willing to screen in the future among those who had never undergone CRC screening.

## Discussion

We present a study that assesses the awareness of and attitudes towards undergoing CRC screening in Lebanon, a model of a developing country with diverse socio-economic levels and standards of living. Our results indicated that only 31.5% and 17.2% of the participants were aware of the warning signs and risk factors of CRC, respectively. These low percentages are in line with previous studies conducted in the MENA region. In these studies, it was found that more than 60% of the Lebanese population had never heard of CRC [3], and about 82%, 85% and 94% of participants from the United Arab Emirates had poor to no knowledge on colorectal cancer risk factors, warning signs and screening methods respectively [11]. It is worth noting here that our study population is not representative of the general population of Lebanon since participants were selected from the largest tertiary medical care center in Beirut, and more than half of them had a university level education. Thus, if our study were to include participants from a more rural areas, the percentages of awareness would be anticipated to be lower than what was observed.

The CRC risk factors that were most commonly recognized were red meat (64%), bowel disease (62%), and smoking (60%). Participants were least aware of diabetes (26%) and no physical exercise (37%) as risk factors, and only 17.2% of the participants were aware of nine or more of the listed risk factors. Our results are in line with previous findings, whereby family history, alcohol, and smoking were the most commonly known risk factors in the Lebanese population, while diabetes and sedentary lifestyle were the least commonly known risk factors [3]. Hence, awareness of dietary risks was higher than awareness of the effect of weight and physical activity, as previously reported in the literature [12,13].

Participants were most commonly aware of anal bleeding and presence of fecal blood (68.2% and 65.3%, respectively) as warning signs for CRC, and were least aware of anemia and fatigue as warning signs (39.3%). Individuals were more likely to be aware of CRC risk factors and warning signs if they knew someone who was diagnosed with CRC. This association was also reported in a number of other studies [3,10,14].

While different studies have demonstrated an increase in awareness of CRC screening with education and employment, our results showed no significant association with these factors [3,10,12,15]. This discrepancy could be a result of adjusting for additional covariates in our regression model, such as; regular physician check-ups and methods of awareness, which proved to be significantly associated with awareness of the necessity of CRC screening and which were not accounted for in those studies. Failing to detect a significant association between university and non-university educated participants and levels of CRC awareness highlights the challenges in raising awareness and care standards in developing as opposed to developed countries.

Moreover, awareness of the necessity of screening was found to be related to awareness of risk factors for CRC and having a family physician as a primary source of knowledge about cancer. This is in agreement with previous reports which indicated that individuals with a greater knowledge and a neutral to favorable attitude towards CRC and its screening protocol were more likely to screen [16]. Other studies showed that the most frequently given reasons for not being up to date with screening included “doctor did not order it” and “unaware of the importance of colorectal screening [17,18].”

Our results showed that 15% of the surveyed sample had previously undergone colorectal cancer screening and 45% of participants reported that they were unaware that they should screen for CRC. This is higher than the screening rate of CRC in Saudi Arabia, which was found to be 8.6% [19], and closer to that in Spain, where 12% of the surveyed population had undergone CRC screening [20]. In fact, the lack of knowledge that one should get screened has been found to be one of the most common barriers to CRC screening [9,14,18,19,21]. Primary care physicians were shown to play an important role in recommending CRC screening and increasing awareness of screening guidelines [10]. This agrees with our study where regular physician checkups were associated with increased willingness to screen. In a study by Lemon et al. patients stated that the absence of strong physician recommendation was a reason behind the low screening rates [22] as discussion of health-related issues would not take place [23]. In addition, recommendations from family, friends or colleagues who have had a positive experience with colorectal cancer by being cured from this disease, are expected to increase patients’ and participants’ compliance to screening [24].

Out of the participants who were unwilling to get screened, 52% reported that they would not get screened because there was no one in the family with CRC, indicating a lack of awareness that CRC most often occurs in individuals with negative family history; this pattern was established by a number of other studies in the literature [14,18]. Our results also indicated that 28% of the participants were unwilling to get screened because they believed that there was no escape from God’s will; A reason that points to a sense of fatalism or futility related to the detection of colorectal cancer. A systematic review of the literature has revealed that fear and fatalism commonly acted as barriers to participation in CRC screening [14]. This attitude likely stems from a false belief that the progression of CRC, as well as other types of cancer, is unpreventable. The majority (60%) of individuals who were unwilling to get screened, however, reported that screening was of low priority, which is in agreement with a study by Bidouei et al. in East Iran [10]. In fact, priority given for CRC screening is linked to the person’s perceived risk for the disease, which itself depends on the understanding of the disease. A low perceived risk is associated with lower preventive measures [25,26,27,28]. Barriers such as cost and inaccessibility to screening centers have been identified in the literature but were not shown to be significant in our study [8,14,18,21].

Among the surveyed participants, 43% were willing to get screened in the future and stated the method they would choose to get screened as such: 65% preferred FOBT, and 54% preferred colonoscopy. These results are similar to what was reported by DeBourcy et al. [29] wherein more than half of the respondents preferred FOBT over colonoscopy. Evaluating the reasons behind these choices elucidates the factors that have highest influence on screening choices, and hence may provide guidance for future efforts to increase screening rates in the Middle Eastern population. The most commonly reported reasons for getting screened by FOBT rather than colonoscopy were: “faster”, “easier”, “less painful” and “less embarrassing”. As for those preferring to get screened by colonoscopy rather than by FOBT, the reasons were: “more accurate” and “recommended by physician”. The results are reinforced by data obtained by Ling et al. [30] where features of the screening test that participants were interested in were linked to the method of screening they choose. Many of the participants, especially those who were unwilling to repeat the screening in the future, chose “pain” as a barrier towards choosing colonoscopy as a screening modality. A possible solution would be the addition of a short interval of time at the end of the procedure, during which the tip of the colonoscope remains in the rectum. It was reported that this practice can help alleviate some of the pain and unpleasant feeling among patients, which in return could encourage them to repeat the screening when needed [31].

Among participants who had never screened for CRC and were unwilling to screen in the future, barriers mostly revolved around misconceptions about cancer in general, and colorectal cancer in specific. Willingness to be screened was higher among individuals who were aware of risk factors for colorectal cancer. This finding lends support to the fact that efforts should be made to increase awareness of CRC warning signs and risk factors in order to increase screening rates.

## Conclusion and Recommendations

We present a novel study that correlates awareness of colorectal cancer risk factors and warning signs to the attitudes toward its screening guidelines and techniques, in addition to unraveling factors that could pose barriers towards undergoing CRC screening. Our generated results are of great importance since they offer proper insight to maximize the impact of future efforts to increase CRC screening rates in Lebanon. First, as the awareness of risk factors for CRC is positively correlated with willingness to get screened, it is important to emphasize the risk factors for CRC in any context where CRC screening is being promoted. It is particularly important to spread awareness about the CRC risk factors and warning signs that people were least aware of, like lack of physical activity, having diabetes, anemia, or fatigue. Second, there should be an increase in public awareness campaigns which emphasize that CRC screening is recommended for everyone and not only for those with family history, since screening leads to early detection, and prevention of disease progression, hence a decrease in mortality rate. Rectifying the misguidance is expected to help people overcome the fear and stigma towards colorectal cancer. Listening to stories of colon cancer survivors, especially from celebrities has been shown to have a positive impact on following screening guidelines [32]. Our results indicated that the majority of the Lebanese population acquired their knowledge about CRC through family and friends and/or media such as internet and television (TV). Hence, launching structured awareness campaigns and dedicating an awareness month for this disease can increase public knowledge about CRC. Advertising for these activities on TV and social media will certainly help reach a wider range of audience with different age groups and backgrounds. Third, our study showed that a lot of value and consideration is given to physician recommendations, emphasizing the role of general practitioners and family physicians in the screening and prevention of colorectal cancer. In this regard, health care systems and authoritative groups should work towards instituting colon cancer awareness as mandatory for each contact with patients. Moreover, awareness campaigns should stress on the pivotal role of primary care physicians in stimulating communication with patients to spread public awareness about the importance of screening and the different screening modalities, and more importantly, to attenuate the stigma of shame and embarrassment that proved to be a major barrier towards CRC screening in our results. Finally, the government should institute a policy of free CRC screening covered by the Ministry of Health after the age of 45 as well as when ordered by the primary care physician, with the hope that this would increase compliance with screening. Early screening and detection lead to decrease in the burden of this disease which in return translates into reduced cost of treatment and improved patient health and wellbeing.

## Additional Files

The additional files for this article can be found as follows:

10.5334/aogh.2437.s1 Appendix 1. Validation of CRC risk factors and warning signs awareness CAM questionnaires.

10.5334/aogh.2437.s2 Appendix 2. Detailed results of the validation of CRC risk factors and warning signs awareness CAM questionnaires.
