# Supplementary material for: Awareness of Colorectal Cancer and Attitudes Towards Its Screening Guidelines in Lebanon
Source: Ann Glob Health. 2019 May 28;85(1):75. doi: 10.5334/aogh.2437 (PMC6634322; doi:10.5334/aogh.2437)
Supplement: Appendix 1. — Validation of CRC risk factors and warning signs awareness CAM questionnaires. [file agh-85-1-2437-s1.pdf]

Appendix 1:

Validation of CRC risk factors and warning signs awareness CAM questionnaires: To validate the Arabic version of bowel cancer warning signs and risk factors awareness questionnaires, we submitted both the original English questionnaires and the Arabic ones to a convenient sample of 21 American University of Beirut's students fluent in both English and Arabic. These participants were asked for oral consent and we explained to them the purpose of the study and the need for validation. Afterwards, they were first given the English questionnaires to fill and later the Arabic versions. The answers were collected on paper questionnaires and entered in Excel 2016 and analyzed using Stata 13 version. The proportion of answers per categories of each variable was generated and the percent agreement with its 95% confidence interval generated to assess the validity of the Arabic questionnaire. Awareness of bowel cancer warning signs was generated as a composite variable from the 9 alarming signs as described above. Individuals knowing a minimum of 7 warning signs were considered knowledgeable and others not. The same process was used to create the risk factors awareness variable, with subjects knowing a minimum of 9 out 12 risk factors considered knowledgeable and others not. Proportion of knowledgeable subjects were generated for the Arabic and English questionnaires, followed by the percent agreement.

The results (Tables 1 and 2 in Appendix 2) showed that the Arabic questionnaires are valid, with good compliance between Arabic and English answers. For subjects' awareness on bowel cancer danger signs, agreements between the Arabic and English questions were all significantly close to perfect, ranging between 0.81 for "Pain in the back passage" to 0.95 for "Anal bleed", "Blood in stools", and "Anemia or fatigue" (P-values < 0.0001) (Appendix 2, Table 1). The overall awareness of warning signs had an almost perfect and significant

agreement between the Arabic and English questionnaires with an overall agreement of 95% (P-value < 0.0001). Agreements close to perfect or perfect were also obtained for most risk factors awareness variables (Appendix 2, Table 2). Percent agreement ranged between 81% for "Eating red meat" to 1 for "Age" and "Drinking alcohol". The lowest percent agreement was observed for the "Presence of bowel disease" with 76% agreement. However, a value of 76% was a considered a substantial agreement as per Landis and Koch classification.[33] The overall awareness of risk factors had 95% overall agreement, a percent agreement significantly close to perfection (P-value < 0.0001) (Appendix 2, Table 2).

Reference

33. **Landis JR** and **Koch GG.** The measurement of observer agreement for categorical data. *Biometrics*. 1977; 159–174. DOI: https://doi.org/10.2307/2529310
