# Supplementary material for: Awareness of Colorectal Cancer and Attitudes Towards Its Screening Guidelines in Lebanon
Source: Ann Glob Health. 2019 May 28;85(1):75. doi: 10.5334/aogh.2437 (PMC6634322; doi:10.5334/aogh.2437)
Supplement: Appendix 2. — Detailed results of the validation of CRC risk factors and warning signs awareness CAM questionnaires. [file agh-85-1-2437-s2.pdf]

Appendix 2:

Table 1: Proportion of answer per categories of warning signs awareness variables and percent agreements with 95% confidence interval between the Arabic and English reference questionnaires.

Warning signs	English questionnaire (ref)		Arabic questionnaire		Percent agreement	95% CI of percent agreement	p-value
	N	%	N	%			
**Anal bleed**					0.95	(0.85, 1.00)	0.000
Yes	13	61.90	14	66.67			
No	5	23.81	4	19.05			
Don’t Know	3	14.29	3	14.29			
**Pain in abdomen**							
Yes	10	47.62	8	38.10	0.91	(0.76, 1.00)	0.000
No	4	19.05	6	28.57			
Don’t Know	7	33.33	7	33.33			
**Change in bowel habits**							
Yes	10	47.62	11	52.38	0.86	(0.69, 1.00)	0.000
No	6	28.57	6	28.57			
Don’t Know	5	23.81	4	19.05			
**Bowel not emptying**							
Yes	4	19.05	4	19.05	0.90	(0.76, 1.00)	0.000
No	9	42.86	9	42.86			
Don’t Know	8	38.10	8	38.10			
**Blood in your stools**							

Yes	14	66.67	15	71.43	0.95	(0.85, 1.00)	0.000
No	5	23.81	4	19.05			
Don't Know	2	9.52	2	9.52			
**Pain in your back passage**							
Yes	5	23.81	6	28.57	0.81	(0.62, 0.99)	0.000
No	9	42.86	9	42.86			
Don't Know	7	33.33	6	28.57			
**Lump in anus**							
Yes	10	47.62	8	38.10	0.90	(0.76, 1.00)	0.000
No	3	14.29	4	19.05			
Don't Know	8	38.10	9	42.86			
**Anemia / fatigue**							
Yes	5	23.81	4	19.05	0.95	(0.85, 1.00)	0.000
No	12	57.14	12	57.14			
Don't Know	4	19.05	5	23.81			
**Unexplained weight loss**							
Yes	8	38.10	8	38.10	0.90	(0.76, 1.00)	0.000
No	8	38.10	8	38.10			
Don't Know	5	23.81	5	23.81			
**Danger signs awareness**							
Aware	1	4.76	0	0.000	0.95	(0.85, 1.00)	0.000
Not aware	20	95.24	21	100.00			

Table 2: Proportion of answer per categories of risk factors awareness variables and percent agreements with 95% confidence interval between the Arabic and English reference questionnaires.

Risk factors	English questionnaire (ref.)		Arabic questionnaire		Percent agreement	95% CI of percent agreement	p-value
	N	%	N	%			
**Age**							
40-year-old	3	14.29	3	14.29	1	--	--
60-year-old	7	33.33	7	33.33			
Unrelated to age	11	52.38	11	52.38			
**Drinking alcohol**							
Yes	6	28.57	6	28.57	1	--	--
No	10	47.62	10	47.62			
Don't know	5	23.81	5	23.81			
**Not eating Fruit and vegetables**							
Yes	5	23.81	5	23.81	0.95	(0.85, 1.00)	0.0000
No	14	66.67	13	61.90			
Don't know	2	9.52	3	14.29			
**Eating red meat**							
Yes	10	47.62	9	42.86	0.81	(0.62, 0.99)	0.0000
No	7	33.33	8	38.10			
Don't know	4	19.05	4	19.05			
**Low fiber diet**							
Yes	8	38.10	8	38.10	0.90	(0.76, 1.00)	0.0000
No	9	42.86	8	38.10			
Don't know	4	19.05	5	23.81			

**Obesity**							
Yes	8	38.10	8	38.10	0.90	(0.76, 1.00)	0.0000
No	7	33.33	7	33.33			
Don’t know	6	28.57	6	28.57			
**Being 70 years old**							
Yes	13	61.90	12	57.14	0.95	(0.85, 1.00)	0.0000
No	7	33.33	8	38.10			
Don’t know	1	4.76	1	4.76			
**Having a relative with CRC**							
Yes	8	38.10	8	38.10	0.86	(0.69, 1.00)	0.0000
No	9	42.86	12	57.14			
Don’t know	4	19.05	1	4.76			
**Absence of physical activity**							
Yes	2	9.52	3	14.29	0.90	(0.76, 1.00)	0.0000
No	16	76.19	16	76.19			
Don’t know	3	14.29	2	9.52			
**Presence of bowel disease**							
Yes	16	76.19	15	71.43	0.76	(0.56, 0.96)	0.0000
No	4	19.05	4	19.05			
Don’t know	1	4.76	2	9.52			

Note: Ref. = reference

Table 2 (Continued): Proportion of answer per categories of risk factors awareness variables and percent agreements with 95% confidence interval between the Arabic and English reference questionnaires.

Risk factors	English questionnaire (ref.)		Arabic questionnaire		Percent agreement	95% CI of percent agreement	p-value
	N	%	N	%			
**Having diabetes**							
Yes	6	28.57	6	28.57	0.90	(0.76, 1.00)	0.0000
No	9	42.86	9	42.86			
Don't know	6	28.57	6	28.57			
**Smoking**							
Yes	8	38.10	8	38.10	0.86	(0.69, 1.00)	0.0000
No	7	33.33	8	38.10			
Don't know	6	28.57	5	23.81			
**Awareness of risk factors**							
Aware	1	4.76	1	4.76	1	--	--
Not aware	20	95.24	20	95.24			

Note: Ref. = reference
